# The dynamics of unstable waves in sea ice

**DOI:** 10.1038/s41598-023-40696-3

**Published:** 2023-08-22

**Authors:** Alberto Alberello, Emilian Părău, Amin Chabchoub

**Affiliations:** 1 School of Mathematics, University of East Anglia, Norwich, NR4 7TJ UK; 2 School of Civil Engineering, The University of Sydney, Sydney, NSW 2006 Australia; 3 Hakubi Center for Advanced Research, Disaster Prevention Research Institute, Kyoto University, Kyoto, 606-8501 Japan

**Keywords:** Fluid dynamics, Physical oceanography, Applied mathematics

## Abstract

Wave and sea ice properties in the Arctic and Southern Oceans are linked by feedback mechanisms, therefore the understanding of wave propagation in these regions is essential to model this key component of the Earth climate system. The most striking effect of sea ice is the attenuation of waves at a rate proportional to their frequency. The nonlinear Schrödinger equation (NLS), a fundamental model for ocean waves, describes the full growth-decay cycles of unstable modes, also known as modulational instability (MI). Here, a dissipative NLS (d-NLS) with characteristic sea ice attenuation is used to model the evolution of unstable waves. The MI in sea ice is preserved, however, in its phase-shifted form. The frequency-dependent dissipation breaks the symmetry between the dominant left and right sideband. We anticipate that this work may motivate analogous studies and experiments in wave systems subject to frequency-dependent energy attenuation.

## Introduction

Arctic and Antarctic sea ice play a prominent role in the Earth system by regulating heat and momentum exchanges over large spatial scales^1–4^. The sea ice properties are intimately linked to ocean wave properties via feedback mechanisms in the marginal ice zone (MIZ)^5–7^ which around Antarctica, fed by intense Southern Ocean waves all year round^8^, extends for hundreds of kilometers^9–11^. Rapid evolution of the polar regions driven by climate change^12–14^ have revived and energised research activities in understanding waves properties and feedback in the MIZ^7,15^, including in the emerging Arctic MIZ^16^.

**Figure 1:**
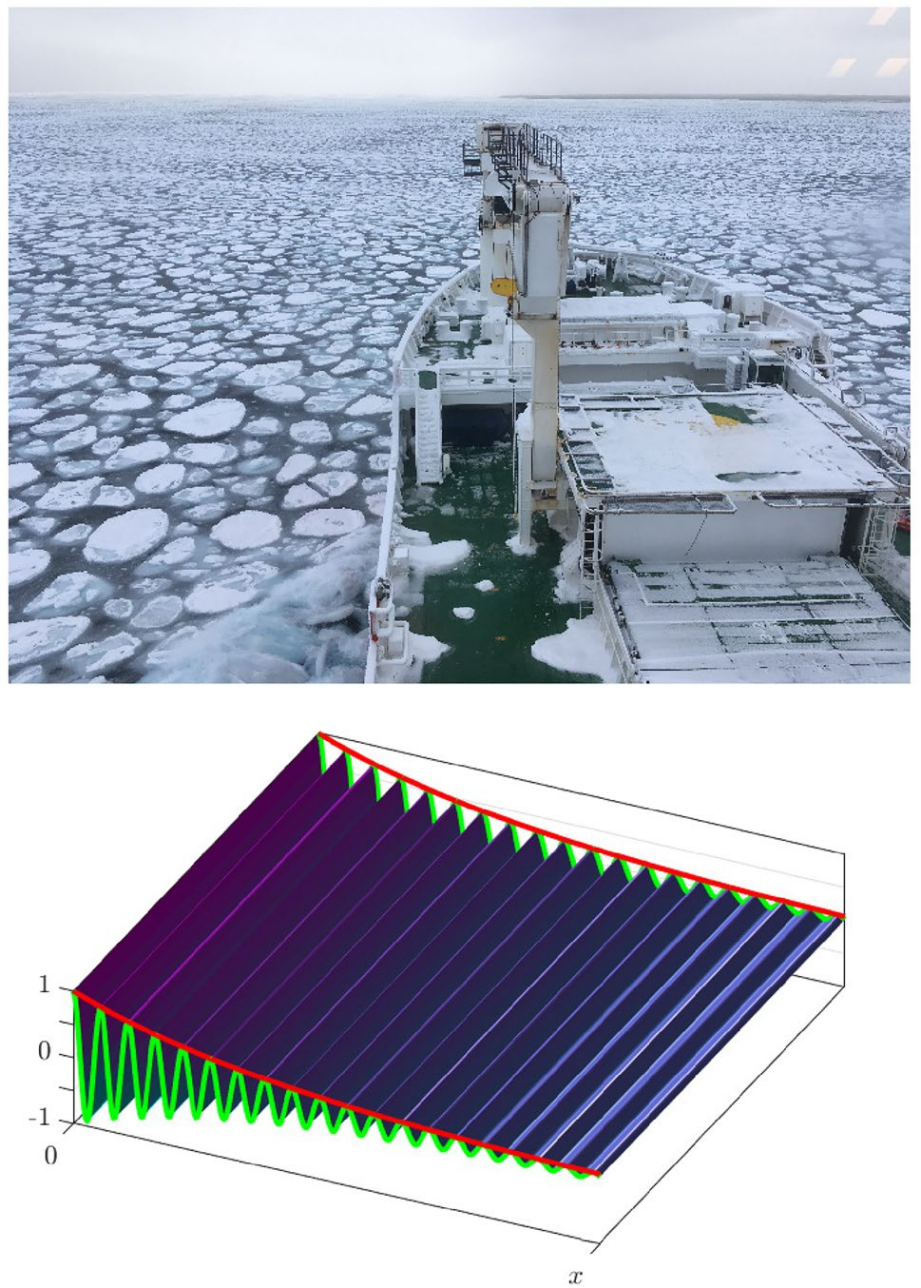
Example of Southern Ocean waves (wave height $\approx 5$ m and peak period $\approx 12$ s) propagating in a MIZ comprised of small ice floes (1–10 m) as seen from the icebreaker S.A. Agulhas II (beam 21.7 m, for visual reference) on the 24 July 2022 at 59$^\circ$S and 1$^\circ$E, and schematic of exponential dissipation for a monochromatic wave of unit amplitude propagating from left to right. In the schematic the green line denotes the surface elevation and the red line the wave envelope which undergoes exponential attenuation with distance.

In the MIZ exterior, where the sea ice cover is a mixture of small floes (much shorter than the wavelength) and interstitial frazil ice^17,18^, as shown in Fig. 1, viscous-like losses have been identified as the main wave attenuation mechanism^19–21^. In the MIZ interior, where floes are larger and comparable to the wavelength, wave attenuation by scattering dominates^20^. At leading order in wave steepness, i.e. the wave nonlinearity parameter, each wave component attenuates exponentially with distance, see schematic in Fig. 1, and at a frequency-dependent attenuation rate^19,22,23^. That is, shorter waves are attenuated faster than their longer counterparts. For a comprehensive review of waves in sea ice we refer the reader to Meylan et al.^19^ and Squire^20^, and references therein.

Narrowband ocean wave dynamics can be accurately described by the nonlinear Schrödinger equation (NLS). One intriguing dynamical phenomenon, which is responsible for the formation of large-amplitude and coherent waves and has attracted the scientific interest since the late 60s, is the modulation instability (MI)^24^. In fact , and in contrast to the linear stability analysis of Stoke waves, the complete growth and decay cycles can be described within the NLS framework^25^. More recently, several studies have been devoted to investigate the effect of wave dissipation on the phase-shifted recurrent MI focusing cycles^26–31^. The latter studies highlight the phase-shifted recurrence in the long-term evolution of nonlinear and unstable waves, when constant, weak, and linear dissipation effects are at play. That said, the NLS can be also adapted to accommodate the influence of sea ice attenuation on the waves by including viscous-like losses as a dissipative term that matches the decay rate of the linear amplitude, as shown by^32,33^. Within this context, it as been shown that it is important to account for the ice-induced frequency dependency in the attenuation of ocean waves in the MIZ^34^.

We will focus our attention on the evolution dynamics in infinite depth regime since most of the sea ice processes are relevant in deep-water regimes. In this case the weakly nonlinear spatial evolution of damped hydrodynamic waves can be described by the d-NLS [e.g^26,34^]:

1 $$\begin{aligned} \psi _x +i \dfrac{k_0}{\omega _0^2}\psi _{tt}+ i k_0^3|\psi |^2\psi = -\mathfrak {D}(\omega ) \psi , \end{aligned}$$

where $\psi$ is the complex wave amplitude, which is evolving along the space co-ordinate *x*, while *t* denotes the time in the frame of reference moving with at the group speed ($t=t'-x/c_{g}$ where $c_g$ is the group speed and $t'$ the time in the fixed frame of reference), *i* is the imaginary unit, *g* the gravitational acceleration, $k_0$ the wavenumber of the carrier wave, $\omega _0=\sqrt{gk_0}$ the angular frequency, and $\mathfrak {D}(\omega )$ the frequency-dependent linear attenuation rate. Eq. (1) is therefore written in the frame of reference moving with at the group speed. We remark that the damping term is introduced using an heuristic approach, and is equivalent to the one introduced in^34^ with the incorporation of a whole range of dissipative parameters while accounting for the group velocity as reference celerity.

Following^19^, the viscous-like wave dissipation due to sea ice follows a frequency power law with a particular feature that higher frequency components undergo stronger attenuation:

2 $$\begin{aligned} \mathfrak {D}(\omega )=\alpha _n\left( \omega _0+\delta \omega \right) ^n, \end{aligned}$$

where we use $\omega =\omega _0+\delta \omega$ in the formulation of the dissipation coefficient to explicitly highlight the difference in frequency between the carrier wave angular frequency $\omega _0$ and other spectral components. The power law exponent *n* depends on the physical mechanisms at play^20^ (e.g. viscous losses^16^, basal friction^35^, scattering^21,36^, overwash^37,38^, floe slamming^39^), and usually falls in the range 2–4^19^. The same applies for the real coefficients $\alpha _n$, which also depends on the same dynamical and complex interactions^19^ and, therefore, on effective sea ice properties (e.g. thickness, density, effective viscosity)^40^. The power law dissipation with exponent $n=3$, as mentioned in Eq. (2), was found to agree well with field observations and the mathematical modelling^19^, and will be therefore considered herein. The exponent *n* is derived from the classical linearised water wave problem when an extra pressure term is added to the free surface dynamic boundary condition to model sea ice. When the pressure term is assumed proportional to the vertical velocity the resulting wave dispersion relation has imaginary part (equivalent to attenuation rate) proportional to $\omega ^3$^19^. Note that the model is equivalent to the one discussed in^34^, but in this case the dissipative coefficients $\alpha$ incorporates all the physical parameters and the evolution of the wave envelope is written in the frame of reference moving at group speed. The model can be easily modified to include other forms of frequency-dependent dissipation.

We investigate the classical MI problem of a monochromatic wave train with frequency $\omega _0$ and initial amplitude $a_{i}$ subjected to initially small symmetric sideband perturbations $a_{i}^l$ and $a_{i}^r$:

3 $$\begin{aligned} \psi _i(t) = a_{i}+a_{i}^l e^{-i \delta \omega t}+a_{i}^r e^{i \delta \omega t}. \end{aligned}$$

Note that no phase shift between the carrier and sidebands is imposed. The initial amplitude of the left and right sideband, $a_{i}^l$ and $a_{i}^r$, respectively, is set as 1% of the carrier wave amplitude, i.e. $a_{i}^l=a_{i}^r=0.01a_{i}$, and the frequency difference as $\delta \omega =0.1\omega _0$. Note that the chosen $\delta \omega$ gives the maximum growth rate of the sidebands in ice free waters^24,41^.

The wave steepness, defined using wavenumber of the carrier wave and the sum of all amplitudes, [e.g.^42^]:

4 $$\begin{aligned} \varepsilon =k_0\left( a+a^l+a^r\right) , \end{aligned}$$

is initially set to 0.1, also defining $T_p=2\pi /\omega _0=12$ s. The corresponding wavelength and wavenumber are $L=225$ m and $k_0=2.8\times 10^{-2}$ $\hbox {m}^{-1}$ respectively. Wave period and steepness are representative of intense storm waves at the edge of the Antarctic MIZ^18^, wave conditions few tens of kilometers away from the sea ice edge and within the MIZ are illustrated in Fig. 1. Besides the conservative case used as a reference, 4 dissipation levels defined by $\alpha _3$ value which span a large range are analysed (summarise in Table 1). The dissipative cases are arbitrarily defined with respect to each other as low, medium, high and very high.

**Table 1:** Dissipation levels in the simulations.

Dissipation	$\alpha _3$ [$\hbox {s}^{3}\hbox {m}^{-1}$]	$\mathfrak {D}(\omega _0)$ [$\hbox {m}^{-1}$]	$\langle \Psi \rangle _{X=445}$ [–]
N.A.	0	0	1.000
Low	7 $\times$ 10$^{-7}$	10$^{-7}$	0.991
Medium	7 $\times$ 10$^{-6}$	10$^{-6}$	0.905
High	7 $\times$ 10$^{-5}$	10$^{-5}$	0.372
Very high	1.4 $\times$ 10$^{-4}$	2 $\times$ 10$^{-5}$	0.136

This work will focus on the physical effects on the unstable wave dynamic when considering frequency-dependent dissipation in the wave modelling to third-order in wave nonlinearity. We particularly show that the shifted MI recurrence is retained, but with a noticeable decrease in the recurrent focusing period with the increase of the sea ice dissipation value. Moreover, the asymmetric damping in wave energy components leads to an instrinsic behaviour of the dominant sidebands in the respective phase space. We anticipate that this study will motivate numerical and experimental studies in several nonlinear wave systems governed by a frequency dependent forced/damped NLS, e.g. optical cavities^43^, nonlinear optics^44^, exciton-polariton Bose–Einstein condensates^45^, plasma physics^46^, and metamaterials^47^.

## Results

### Spatial evolution and recurrence

**Figure 2:**
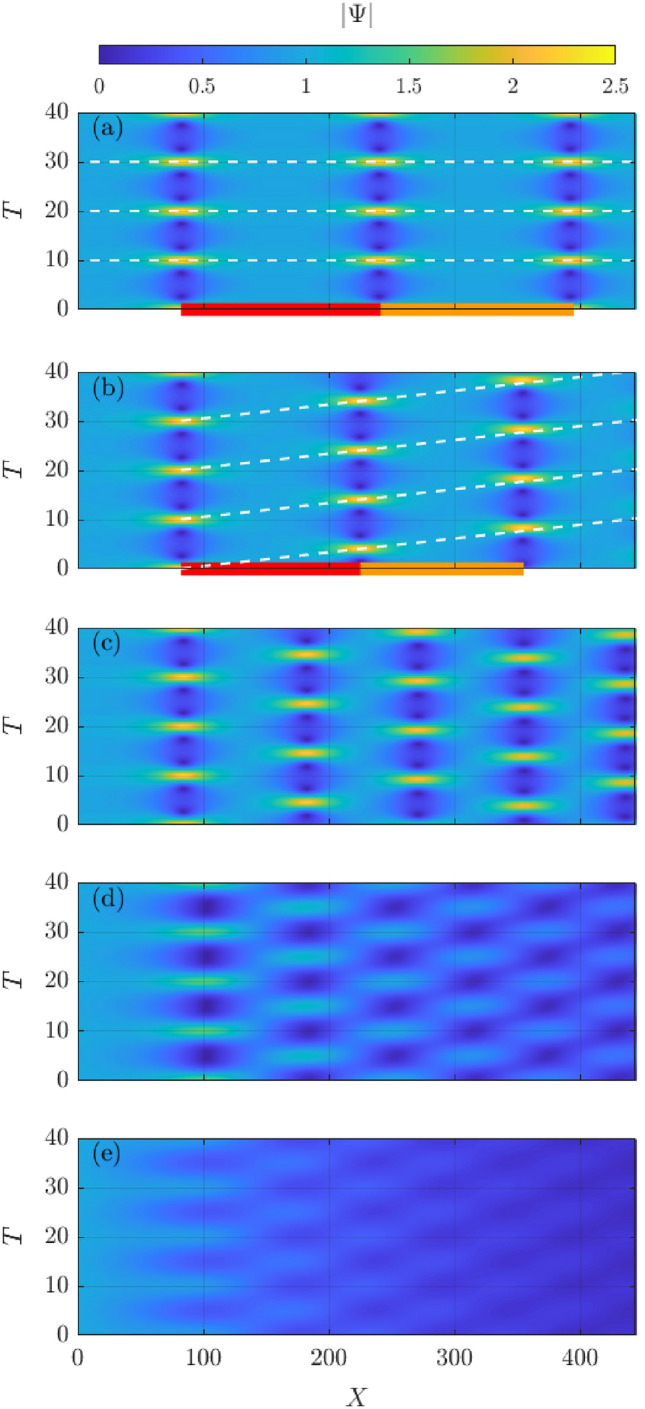
Spatial evolution of the unstable wave envelope in the time domain for increasing dissipation (top to bottom) from none (**a**) to very high (**e**). The corresponding dissipation values $\mathfrak {D}$ for the carrier wave are reported in Table 1. In panels (**a**), i.e. the conservative B-Type breather, and (**b**), i.e. the dissipative A-Type breather, the recurrence distances (coloured bars at the bottom) and the recurrence patterns (dashed white lines) are highlighted.

The spatial evolution of the unstable dimensionless, normalized, envelope $|\Psi |=|\psi |/a_{i}$ in absence of dissipation, i.e. when considering the conservative case, is shown in Fig. 2a. Indeed, this is a well-anticipated and know intrinsic dynamic, which involves recurrent focusing cycles with same wave amplification factor along the dimensionless space co-ordinate $X=x/L$. Moreover, when considering adjusting the wave packet motion with respect to the group speed, all periodic wave amplification maxima occur at the same dimensionless time $T=t/T_p$. This corresponds to the pulsation dynamics of a B–Type doubly-periodic breather^48^ for which the recurrence distance is $\approx$160 wavelengths for the chosen wave parameters. Indeed, this is in good agreement with the theoretical value predicted using:

5 $$\begin{aligned} r=\dfrac{1}{2k_0\varepsilon ^2}\ln {\left[ \dfrac{8}{3}\left( \dfrac{2a}{a^l+a^r}\right) ^4\right] }. \end{aligned}$$

When accounting for a gradual increase of dissipation, as defined by the parameter $\mathfrak {D}$, phase-shifted focusing recurrence, which is a characteristic A–Type periodic feature^26,49^, emerges. The evolution of the respective wave packets are shown in Fig 2b–e. This occurs already for very small values in wave dissipation. Moreover, the distance between the cycles diminishes compared to the conservative case, with the shortening being more evident for growing degrees of dissipation for low and medium dissipation. The first pseudo-recurrence cycle is 150 wavelengths for low dissipation and 130 wavelengths for medium dissipation. The picture is more complex in the two most dissipative cases in which the length of the first pseudo-focusing cycle increases marginally, while immediately followed by the next slight wave focusing. We attribute this behaviour to the rapid decrease of wave amplitude with the increase of wave dissipation, and thus the lower wave nonlinearity results in a slower growth-decay cycle for the MI. This can be also traced in Eq. (5) in which the wave steepness appears in the denominator. As such, the rapid energy loss contributes to the increase of the recurrence period.

By using as a boundary condition the numerical results at the end of each pseudo-recurrence cycle we can obtain an updated value for the recurrence length from Eq. (5). Nevertheless, we note that the predictive ability of Eq. (5) deteriorates at each cycle and for increasing degree of dissipation in low and medium dissipation. The formula usually underpredicts the recurrence length compared to the numerical simulations, e.g. the recurrence length of the third cycle is underpredicted by $\approx 10\%$ in low dissipation and by $\approx 30\%$ in medium dissipation. For the case of high dissipation, the prediction of the recurrence length in fact improves. Compared to the numerical simulations, the pseudo-recurrence length predicted using Eq. (5) is shorter by $\approx 10\%$ at the third cycle and longer by $\approx 20\%$ at the subsequent cycle. This underlines the fact that the wave prediction become unreliable in the case of very high dissipation.

**Figure 3:**
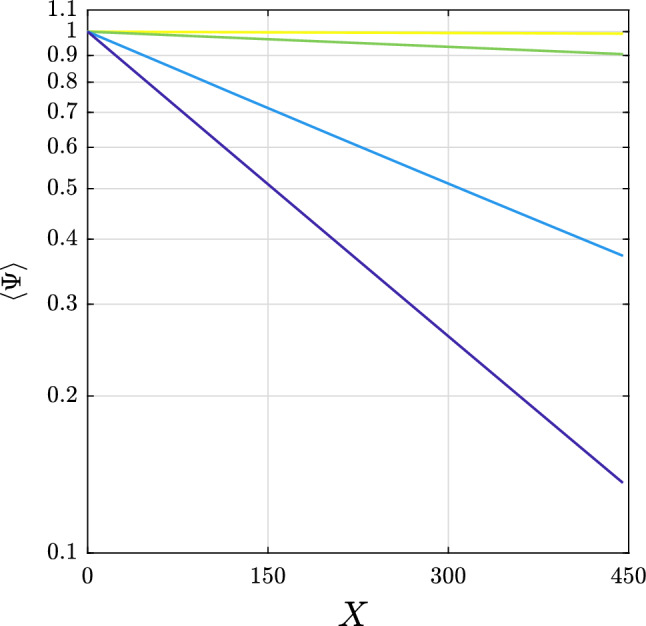
Spatial evolution of the mean wave amplitude in logarithmic scale for the dissipative cases, from low (yellow) to very high (blue), as listed in Table 1.

The spatial evolution of the mean wave amplitude $\langle \Psi \rangle$, computed with respect to the time variable, for the different dissipation levels is summarised in Fig. 3. Note that the vertical axes is in logarithmic scale to highlight deviation from the benchmark exponential decay. In the conservative case, energy is naturally conserved along the propagation in the in space coordinate, i.e. $\langle \Psi (X)\rangle =1$. With the increase of the level of the exponential dissipation rate, i.e. the negative slope is linear in Fig. 3, the mean wave amplitude at the end of the computational domain is summarised in Table 1. These values are within 1% of the energy level of the carrier wave component subjected to linear attenuation, i.e. $\exp {[-\mathfrak {D}(\omega _0)x]}$. Therefore, the presence of modulation instability cycles does not alter the overall energy that is carried by waves in the dissipative sea ice domain. The negligible difference contrasts with simulations for random waves in which the nonlinear cases remained significantly more energetic in the sea ice cover compared to corresponding linear cases and the energy decayed less than exponentially [cf.^34^]. However, it is worth noting that for a continuous spectrum the carrier wave component progressively shifted to longer wave period^50^ which are also less dissipative. On the other hand, the three wave system has a more symmetric spectrum and the period of the carrier wave component remains constant during the long-term evolution in space. It is also noteworthy that for the lowest dissipation level, at the end of the spatial domain in our numerical simulations (approximately three recurrence cycles), the mean wave amplitude is reduced by less than 1%. Nonetheless, the spatial pattern of the recurrence cycles still becomes phase-shifted, see Fig. 2b.

### Wave amplification

**Figure 4:**
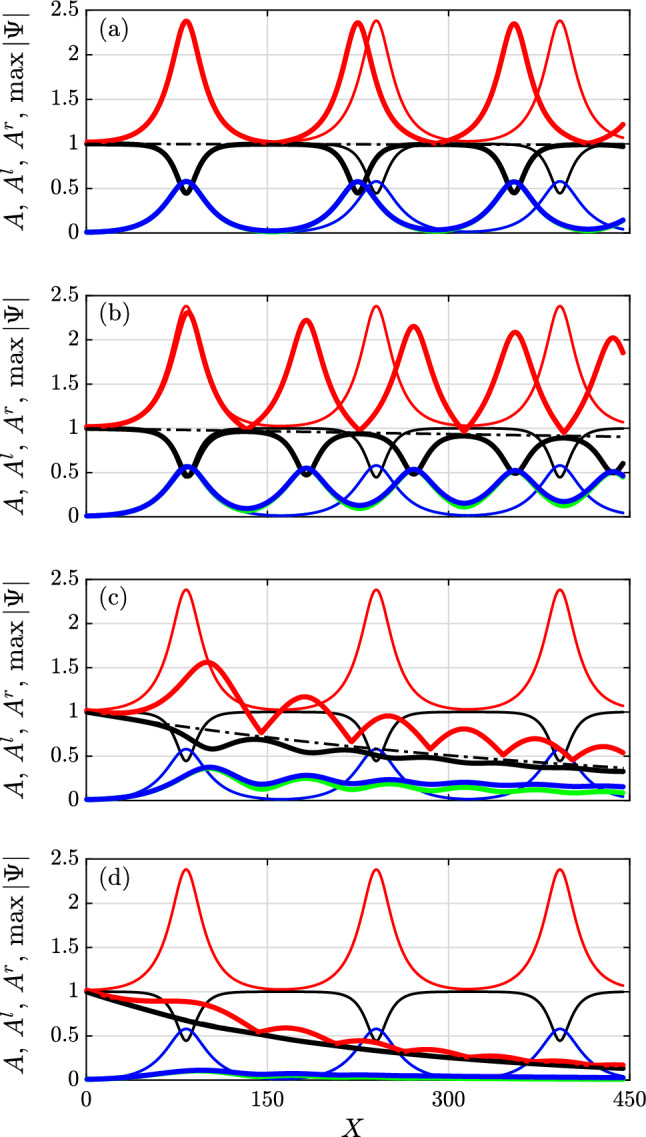
Spatial evolution of the wave amplitudes for low to very high dissipation (top to bottom) for the carrier peak energy (black), left (blue) and right sideband (green), and the maximum amplitude (red). Thin lines denote the conservative case. The dash-dotted line denotes linear decay of the carrier wave amplitude component.

In each of the simulations performed, we tracked the maximum amplification in space, irrespective of time, i.e. $\max |\Psi |$ shown as thick red line in Fig. 4, and compared to the conservative case, depicted in thin red line in Fig. 4. As shown in the surface plots in Fig. 2, the shortening of the recurrence cycle is evident even for low dissipation and as a result the maxima occur at location different from the ones for the conservative case but amplification is almost unaltered. Strikingly, in the medium dissipation case, see Fig. 4b, the spatial frequency of local maxima is almost doubled compared to the conservative case, i.e. the locations in which rogue waves occur, i.e. $\max |\Psi |\ge 2$, is increased for medium level of dissipation. On the first cycle of recurrence the maximum amplification is only slightly diminished and at the end of the numerical domain (after 5 cycles of phase-shifted recurrence) the maximum amplification is $\approx 2$. For greater levels of dissipation, that is, high and very high and as in Fig. 4c–d, the shortening of recurrence cycle is even more pronounced. In the high dissipative case, only the first two cycles of phase-shifted recurrences have amplification larger than one, since the first cycle has an amplification $\approx 1.5$. For very high dissipation, the damping is predominant and the amplitude amplification never exceeds one.

### Sidebands dynamics

The evolution of the carrier wave ($A=a/a_{i}$) and first left and right order sideband $\bigg (A^l=a^{l}/a_{i}$, $A^r=a^{r}/a_{i}\bigg )$ is also shown in Fig. 4. All the cases show the energy exchange from the carrier to the sidebands during the growing phase of the MI cycle and the opposite in the decaying phase. The frequency dependent dissipation would imply that the left (right) sideband undergoes 30% lower (greater) dissipation than the carrier. That said, the difference is small but noticeable only in the high dissipation case difference, in all the other cases the difference is negligible, i.e. the blue and green lines overlap. For low dissipation, only an accelerated cycle in the dynamics of the carrier and the first order sidebands is observed but their amplitude remain almost unaltered compared to the conservative case (thin lines in Fig. 4). For medium dissipation the sidebands never return to the initial amplitude level but at the end of each decay cycle are more energetic, i.e. each local minimum has a higher value of the previous one. For high dissipation rate a similar behaviour is noted, but the higher dissipation rate means that the sidebands are eventually damped. In the high dissipative case, the energy exchange between the carrier and the sidebands is almost suppressed after the first cycle, and the carrier band decays exponentially (the thick black line overlaps the dash dotted line in Fig. 4d).

To study the dynamics of the first order left sideband we construct its phase space diagram as^51^:

6 $$\begin{aligned} \left\{ \eta _{x}^l,\eta _{y}^l\right\} = \left\{ \dfrac{\left| a^{l}\right| ^2}{\left| a_{i}\right| ^2}\cos {\Delta \phi ^{l}}, \dfrac{\left| a^{l}\right| ^2}{\left| a_{i}\right| ^2}\sin {\Delta \phi ^{l}}\right\} , \end{aligned}$$

where $\Delta \phi ^l$ denotes the phase difference between the carrier wave mode and the dominant left sideband, i.e. $\Delta \phi ^{l} = \phi -\phi ^{l}$. The phase space diagram of the right sideband is obtained in similar manner.

**Figure 5:**
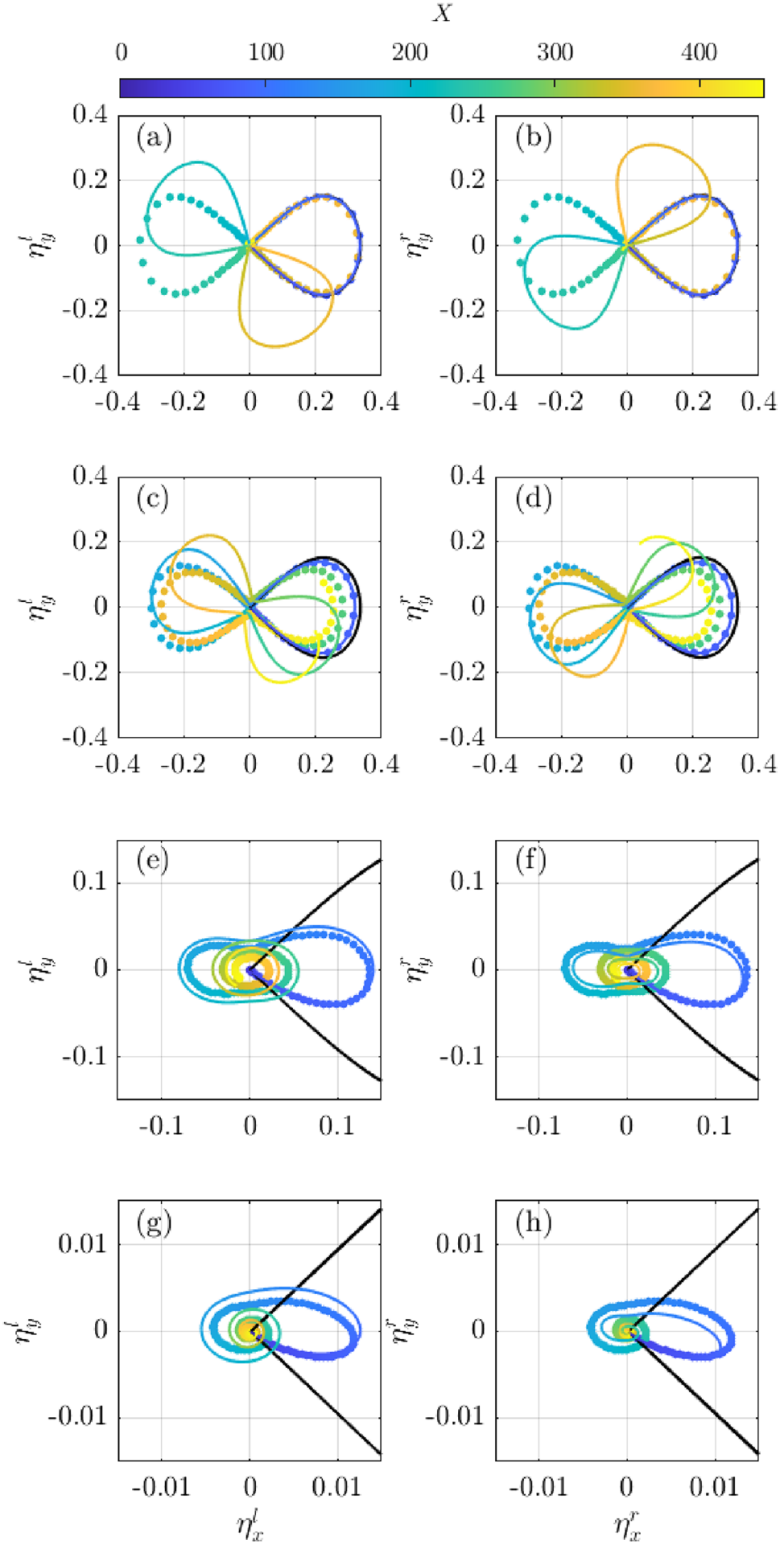
Phase space diagram of the left (**a**, **c**, **e**, **g**) and right sideband (**b**, **d**, **f**, **h**) for low to very high dissipation (top to bottom). The black lines depict the conservative case and the dotted line the frequency independent dissipation. Note that the axis’ limits change in each panel.

Figure 5 reports the conservative case (in black) in which the subsequent cycles of the MI repeat identically in time, i.e. they repeat the same track, and are confined in the right side of the phase space diagram (right and left sideband behave exactly in the same way). For the selected initial condition, the trajectory in the phase space diagram is confined within the separatrix, and recurrence cycle are in-phase. This is consistent with the expected B–Type recurrence. For low to medium dissipation (Fig.5a–d), trajectories switch to an outer trajectory (shaped like an eight) and result in phase-shifted recurrence cycles, i.e. A–Type recurrence. Subsequent cycles for the left (right) sideband move clockwise (anti-clockwise) and at a higher degree for low dissipation. For frequency-independent attenuation no rotation of the main axes in the phase space diagram is observed and the left and right sideband behaves in the same manner, see dotted line in Fig. 5a–d. For high to very-high dissipation the switch to an eight-like shape of the trajectory that spans the left and right hand side of the phase space is also observed, but subsequent loops rapidly degenerate into spiralling cycles due to the substantial attenuation, see Fig. 5e–h.

**Figure 6:**
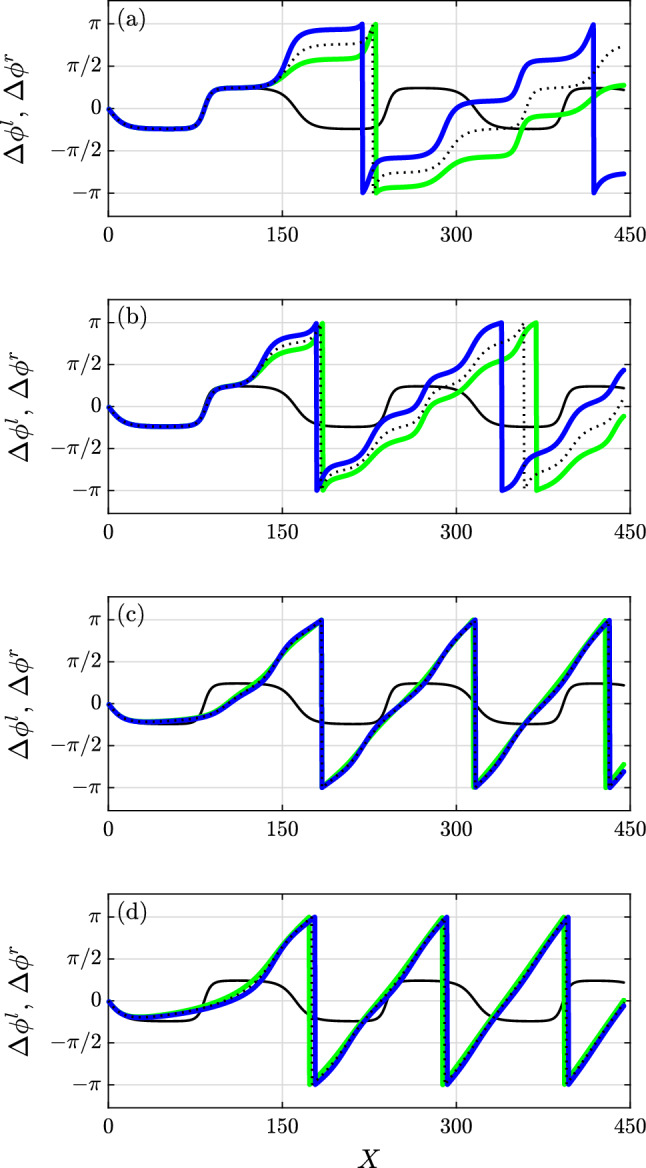
Phase-difference for low to very high dissipation (top to bottom) for the left (blue) and right sideband (green). The black line shows the conservative case and the dotted line the frequency independent dissipation.

The different behaviour of the frequency-dependent and frequency-independent and constant attenuation is clearly shown by examining the phase difference between the sidebands and the carrier, see Fig. 6. For the conservative case, i.e. the case of zero dissipation, the phase difference is the same for the dominant left and right sideband and confined between $-\pi /2$ and $\pi /2$, see continuous black line in Fig. 6. For the frequency-dependent dissipation left and right sideband span angles between $-\pi$ to $\pi$ and behave differently, particularly, for low dissipation levels (Fig. 6a), with the right sideband lagging behind the left one in the rotation. It is worth noting that for frequency-independent attenuation, as denoted by dotted line in Fig. 6, the right and left sideband behave the same and have an intermediate behaviour between the sidebands in the frequency dependent case. Differences between the right and left sidebands in the dissipative case tend to disappear for the higher attenuation levels, see Fig. 6c–d.

## Conclusions

The classical dynamics of the modulational instability is studied in the presence of a frequency-dependent attenuation, this is in contrast with previous works in which forcing and damping were considered frequency independent, e.g.^26,29,31,51^. Nevertheless, similarly to previous works, a small dissipation is capable of altering the recurrence cycle from a B–Type (non-shifted) to A–Type (shifted) breather-type evolution. The most important effect of the differential attenuation rate modifies the dynamics of the left/right sidebands and there is a lag between the two. However, compared to frequency-independent attenuation, differences in energy and recurrence pattern appear unaffected. The large initial nonlinearity of the system triggers pseudo-recurrence cycles with large amplification of the initial sea state, particularly, for low and medium dissipation. Interestingly, for medium dissipation rates the shortening of the cycle means that there are more instances in the domain with amplifications greater than two, compared to the conservative case. For comparison, random sea states, see Ref.^34^, reverted towards Gaussian statistics in presence of dissipation.

Despite the complex dynamics in the nonlinear evolution, the total energy decays in agreement with linear theory, this contrasts the random waves (cf.^34^) in which for large dissipation make a considerable difference between the linear and nonlinear case. This difference is attributed to the shift of the peak frequency in a continuous wave spectrum, whereas for the classical MI case the carrier wave frequency remains unshifted.

Ultimately, the results show that an energetic swell system propagating in the Arctic and Antarctic sea ice can generate exceptionally large waves where none were expected, especially, when dissipation is mild to medium, and could cause unexpected hazardous conditions for vessels operating in the marginal ice zone. The present research highlights the need to further explore the dynamics of high-order NLS-type systems for broadband processes and in the presence of a frequency dependent damping, which could be applied to other physical systems, e.g. nonlinear optics, Bose–Einstein condensates, and metamaterials, in addition to hydrodynamics waves.

## Methods

### Benjamin–Feir index and recurrence distance

The Benjamin–Feir Instability index^52,53^ is

7 $$\begin{aligned} {BFI=\dfrac{\sqrt{2}\varepsilon }{\delta \omega /\omega _0}}. \end{aligned}$$

The instability condition for the framework evolving in time is $0<K<2\sqrt{2}k^2a$, so the one in space becomes $0<\Omega <\sqrt{2}ka\omega$, since ${\Omega }/{\omega }={K}/{(2k)}$. That is, for the same initial long-wave perturbation and assuming a typical exponential energy attenuation under the sea ice the following holds:

8 $$\begin{aligned} BFI^{SI}(x)=\dfrac{\sqrt{2}\varepsilon (x)}{\delta \omega /\omega _0}=\dfrac{\sqrt{2}\varepsilon }{\delta \omega /\omega _0}\exp {(-\mathfrak {D}x)}. \end{aligned}$$

For the chosen wave properties the sea ice Benjamin–Feir index at the ice edge $BFI^{SI}$ is 1.41, i.e. $BFI^{SI}(x=0)=1.41$, for all considered $\mathfrak {D}$ values. This is a sufficient condition to trigger growth-decay cycles, typical to the MI^54,55^.

The theoretical recurrence period for the growth and decay cycle of the unstable wave train is given by^56^. In the framework of a wave propagating we apply the canonical transformation using the group velocity, to obtain the recurrence length (Eq. 5)). Compared to^56^, the ratio between the carrier and the sidebands is modified to account for possible asymmetry in their amplitude. For the chosen boundary conditions the recurrence cycle corresponds to 155 wavelengths using the carrier wavelength *L* as normalising factor.

### Dissipation levels

Various level of dissipation on unstable wave dynamics are analysed in this work: from the conservative case (used as benchmark) to very high attenuation values. Recalling that $n=3$ is used as the power law exponent for the dissipation, the corresponding scaling parameter $\alpha _3$ are reported in Table 1. The linear attenuation rate for the dominant wave frequency is also given, i.e. $\mathfrak {D}(\omega _0)=\alpha _3\omega _0^3$. The dissipation lengthscale can be expressed by the ratio $k/\mathfrak {D}(\omega _0)$ and ranges from infinity for the conservative case, i.e. $\mathfrak {D}=0$, to approximately 1400 wavelengths for the very high dissipative case.

It is worth noting that dissipation scales $\omega ^3$ in our model, therefore the carrier wave component and the sidebands are subjected to different attenuation rates. In particular, by using $\delta \omega = 0.1\omega _0$, we obtain that the left sideband attenuates at a rate $0.73\mathfrak {D}(\omega _0)$ and the right sideband at a rate $1.33\mathfrak {D}(\omega _0)$. Therefore, for sidebands with the maximum growth rate and cubic attenuation with respect with frequency, the difference in attenuation rate between the left and right sideband is almost twice stronger, when considering $\mathfrak {D}(\omega _0+\delta \omega )/\mathfrak {D}(\omega _0-\delta \omega )=1.83$.

### Numerical solution

The d-NLS (1) is solved numerically, by advancing $\psi (x,t)$ in space using the fourth order Runge-Kutta method. A time-periodic domain which encompasses a full cycle of modulation, including 10 wave periods for the chosen $\delta \omega$, is used. As such, this makes an efficient and accurate computation of time derivatives in the Fourier domain possible. The frequency-dependent dissipative term can be computed in Fourier space in a straightforward manner using the Fourier and inverse Fourier transform ($\mathcal {F}$ and $\mathcal {F}^{-1}$ respectively) as the following:

9 $$\begin{aligned} \mathfrak {D} \psi = \alpha _3 \mathcal {F}^{-1} \left[ \omega ^3\mathcal {F} (\psi )\right] . \end{aligned}$$

The time domain is discretized using $2^6$ elements and the resulting timestep is $\delta t=$1.875 s. Thus, $T/\delta t=6.4$ and the unstable wave envelope is propagated over 100 km in space using $\delta x = 1$ m (in dimensionless form the computational domain corresponds to $X=x/L=445$ wavelengths). Temporal and spatial resolutions guarantee numerical stability.

## Data Availability

All data generated or analysed during this study are included in this published article.
